# Obesity and reproduction: a study to determine how effectively medical education enhances awareness of the reproductive risks related to obesity

**DOI:** 10.5935/1518-0557.20170059

**Published:** 2017

**Authors:** Alice S. Rhoton-Vlasak, Kay Roussos-Ross, Girard M. Cua, Erica L. Odera, Tracy A. Irani, Terrie Vasilopoulos

**Affiliations:** 1 Obstetrics and Gynecology, University of Florida, Gainesville, FL 32610, USA; 2 Department of Family Medicine Residency, University of South Florida College of Medicine, Tampa, FL 33612, USA; 3 PIE Center/IFAS, University of Florida, Gainesville, FL 32610, USA; 4 Department of Anesthesiology and Orthopaedics and Rehabilitation, University of Florida, Gainesville, FL 32610, USA

**Keywords:** obesity, reproduction, fertility

## Abstract

**Objective:**

To explore awareness of the reproductive versus the medical risks of obesity in a medical and non-medical college educated population.

**Methods:**

An exploratory prospective research design was used. A 26-question online survey was developed and offered to a sample of medical students/residents (n=325) and non-medical college students (n=102). The data were analyzed using Graph Pad software.

**Results:**

102 non-medical undergraduate students (28% male and 72% female) and 325 resident physicians and medical students (46% male, 47% female, 7% unspecified) responded. Both groups reported higher awareness of the general risks of obesity as compared to the reproductive risks. As expected, lay students reported less awareness of female reproductive issues as compared to the medical group (all *p*-values <0.01). Over 90% of respondents would be motivated to lose weight before pregnancy if they knew of these risks, with more than half planning to have children in the future.

**Conclusion:**

This exploratory study found that despite having at least a college education, the populations studied had relatively low levels of awareness of obesity-related reproductive risks. The medical population had much more knowledge about the other health risks of obesity. The survey provided initial data that might be used to consider knowledge gaps and strategies for engaging and educating medical trainees and the public about the reproductive risks of obesity.

## INTRODUCTION

Obesity is one of the most common healthcare problems in women, but the implications relative to reproduction and pregnancy are unrecognized, overlooked, or ignored because of the lack of specific evidence-based treatment options (ACOG, 2015). In the United States, approximately one third of adults are obese with a 50% rate of overweight and obesity among women of reproductive age (Flegal *et al.*, 2012).

There are significant reproductive and pregnancy-related health risks, which impact the mother, fetus, and neonate. Fetal programming may occur and impact subsequent generations (Artal & Flick, 2013). Complications of pregnancy associated with obesity include antepartum-elevated birth weight, gestational diabetes and hypertension, preeclampsia, increased risk of miscarriage, intrapartum-cesarean delivery, failed induction, failed trial of labor, operative complications, shoulder dystocia, postpartum depression, hemorrhage, wound infections, and endometritis. Fetal risks include an increased risk of congenital abnormalities, macrosomia, fetal growth restriction, and stillbirth. Recent data from a review and meta-analysis also indicated healthcare providers should be aware of the fact that obese women who become pregnant are more likely to experience elevated antenatal and postpartum depression than normal weight women (ACOG, 2015; Molyneaux *et al.*, 2014).

A review of data by Lash & Armstrong (2009) determined that the increasing rates of obesity in the United States and the health implications of excess weight in the reproductive process were severe enough to warrant an increase in educational efforts. Improved obesity awareness and education might encourage women to achieve and maintain a healthy weight, specifically when they consider the possibility of having children. Overall, obesity increases the health risks and complications related to reproduction and pregnancy for the mother and baby (ACOG, 2013), thereby making preconception awareness and counseling of utmost importance.

Practitioners across medical specialties should be aware of the reproductive health risks, so that at any point of medical contact counseling and health interventions can be initiated. Pregnancy, with its enhanced medical provider contact, is the ideal time to educate patients on their health and the health of their infants; pregnancy itself and the maternal drive to reproduce can serve as an ideal health "Teachable moment" (Phelan, 2010). It is also important that the general public be aware of the risks so they can focus on healthy lifestyle choices and trying to normalize their weight before conception in order to optimize the health of their future offspring. The American College of Obstetricians and Gynecologists (ACOG) in December 2015 enlisted additional healthcare practitioners beyond obstetricians to offer specific expertise related to obesity management, especially in the pre-pregnancy planning period (ACOG, 2015). Cordozo *et al.* found that among women with infertility, who have much more contact with healthcare providers during the infertility treatment, there was limited knowledge of reproductive outcomes affected by obesity (Cardozo *et al.*, 2012).

The goal of this study was to conduct a survey to explore awareness of the reproductive risks of obesity in a college-educated medical and non-medical population, hypothesizing that the medical population would have greater levels of awareness of obesity-related health risks, but not necessarily about the reproductive risks. Information from this survey might assist weight loss counseling and public health campaigns, much like tobacco cessation programs, as obesity is the second leading cause of preventable death in the United States, after tobacco.

## MATERIAL AND METHODS

The pilot study was carried out in the form of an online anonymous survey approved by the local IRB. The survey study population included 1215 non-medical university students and 1209 medical students and resident physicians, both populations from a Southeastern University. The response rates from each of the groups were 8.6% (102) and 29.3% (325), respectively. The participants were invited via e-mail to participate in an online survey that inquired about their knowledge of health and specific reproductive risks of obesity. The survey also gathered information on perceived behavioral intentions related to obesity. The survey included 26 questions and is listed in Appendix 1. The survey was designed to study awareness of who might be obese, whether participants were aware of general health risks and, more specifically, reproductive system and pregnancy-related health risks of obesity. The study population was chosen to investigate whether having higher education, college education or higher would result in an increase in awareness of obesity-related risks. The study further investigated whether participants with medical knowledge, i.e. medical students and resident physicians, had greater awareness levels over all general and reproductive health risks related to obesity.

### Data analysis

Analyses were conducted using software package GraphPad. Continuous and scale measures were reported as means and standard deviations, and categorical measures were reported as proportions (%). To assess differences between lay students and medical students, two-tailed, unpaired Student's t-tests were used for scale responses and z-tests were used for proportions. *p*<0.05 was considered statistically significant.

## RESULTS

The survey was sent electronically to non-medical university upper level undergraduate students (n=1209), all medical students, and all resident physicians regardless of training level or specialty (n=1215). Of all contacted individuals, 102 and 325 in each respective group responded. The lay group comprised students from the College of Agriculture and Life Sciences, which includes three pre-professional health majors, while the medical group consisted of medical students and resident physicians. Sixty-five percent of the non-medical and 77% of the medically educated respondents were under 30 years of age, therefore in a group that may still be considering reproduction in the future. The lay group was 28% male and 72% female, while the medical respondents were 46% male and 47% female. Seven percent of respondents in the medical group did not specify their gender.

Overall, both groups were aware of the general obesity-related health risks, with greater knowledge in the medical group. Ninety-seven percent and 100% of respondents knew of the connection between Type 2 diabetes and obesity. Table 1 shows levels of awareness for obesity-related reproductive issues. In both groups the reported data sets have missing values. In these questions on tables, the "N" is not equal to the total group responding. For nearly all female reproductive issues, lay students reported being less aware compared to medical students (all *p* values <0.01). The only exception was awareness of obesity-related reproductive issues in males, with both groups reporting lower awareness.

**Table 1:** Awareness of Obesity-Related Reproductive Issues.

Obesity-Related Questions	Non-medical			Medical			
	N*	M**	SD***	N*	M**	SD***	*p* value
How aware of obesity health related risks are you?	102	3.61	.58	325	3.80	.42	0.0004
How aware of reproductive issues due to obesity in women are you?	97	2.75	.87	287	3.00	.79	0.009
How aware are you of risks to the baby in utero due to maternal obesity?	97	2.51	.95	285	3.01	.86	<0.0001
How aware are your of risks to the baby after birth due to maternal obesity?	97	2.32	.97	289	2.88	.95	<0.0001
How aware of reproductive issues related to obesity in males are you?	93	2.39	.91	286	2.43	.97	0.726

*p*<0.05 is considered statistically significant.

Note:  1 = Unaware, 2 = Somewhat unaware, 3 = Somewhat aware, 4 = Aware.

* N = Number (Total N in each group may be less than 102 or 325 due to missing values on these items);

** M = Mean;

*** SD = Standard deviation.

Table 2 shows awareness levels for female obesity health risks. Percentages may not add up to 100% as there were missing values (non-responses) for many categories. Percentages and *p*-values are based on the total "N" of each group. Medical personnel had more awareness, but often less than 50% knew of elevated risk of ovarian cancer, early neonatal death, uterine cancer, stillbirth or birth defects. Neither sample, however, achieved higher than 70% awareness on any given issue, with some issues (such as increased susceptibility to certain cancers and early neonatal death) reaching just 20%-30% awareness. In comparing the groups, the lay group reported being less aware that female obesity increases the risk for several serious health issues, including uterine (*p*<0.0001), ovarian (*p*=0.0012), and postmenopausal breast cancer (*p*<0.0001), as well as pre-eclampsia (*p*=0.002).

**Table 2:** Awareness of Female Obesity Health Risks.

Before taking this survey, were you aware that female obesity (as defined by a body Mass Index over 30) increases the risk for:	Non-medical**		Medical**		*p* value
	%Yes	%No	%Yes	%No	
Uterine cancer	20.6	75.5	47.4	40.9	<0.0001
Ovarian cancer	28.4	68.6	46.2	41.5	0.0012
Early neonatal death	28.4	65.7	37.5	49.2	0.0873
Stillbirth	31.4	63.7	40.3	47.1	0.101
Postmenopausal breast cancer	33.3	62.7	55.4	33.2	<0.0001
Increased birth defects	42.2	53.9	44.3	43.7	0.704
Heavy menstrual bleeding	45.1	50	53.8	34.2	0.1188
Less responsiveness to fertility treatment	51.0	44.1	51.7	36.6	0.897
Pre-eclampsia	52	43.1	64.6	23.4	0.02
Higher rate of miscarriages	52.9	42.3	54.2	34.8	0.818
Entering puberty at a younger age	59.8	35.3	69.8	19.1	0.056
Cesarean section	59.9	39.2	68.3	20.6	0.112
Menstrual period irregularity	66.7	28.4	69.5	19.1	0.589

* *p*≤0.05 is considered statistically significant;

** Percentages may not add up to 100% if not all respondents answered these questions.

Fortunately, despite these differences in awareness between lay respondents and medical students, 92% and 95%, respectively, of individuals responding said that they would be motivated to change their behavior and attempt to lose weight before conceiving after they knew of the excess risks. Sixty-five percent and 85% claimed they planned to have children in the future. Over half in each group felt the best source of information about obesity and reproductive risks should come from physicians (77% and 78%), and 58% and 60% thought it should come from public media. Most surprising was the finding that both medical and non-medical respondents were very aware of obesity-related health risks, but only somewhat unaware of obesity-related reproductive, pregnancy, and fetal risks.

## DISCUSSION

This survey revealed a gap in knowledge regarding the reproductive and pregnancy-related health risks of obesity in an educated medical and lay population. Though there were high levels of knowledge on the general health risks of obesity, the decreased awareness of the reproductive risks is a significant finding, particularly for our medically educated population. Since the medically educated group will be a future primary source of counseling for obesity prevention and management in their patients, it becomes necessary to explore these findings and discover possible opportunities for education and innovation on this topic.

The need for increased education regarding reproductive health and obesity has been demonstrated in other studies. For example, Phelan *et al.* explored the topic of patient education with regards to weight gain and management in pregnancy. They found that less than half of the participants reported receiving weight gain advice from a practitioner (Phelan *et al.*, 2011; Stengel *et al.*, 2012). Furthermore, 22.2% of the overweight and obese women who did receive counseling were told to expect pregnancy weight gains that exceeded the Institute of Medicine guidelines (Phelan *et al.*, 2011). Excessive weight gain during pregnancy is not advisable, as it leads to increased rates of obesity, as well as worsening of already-existing obesity due to post-pregnancy weight retention. In turn, post-pregnancy weight gain has been linked to obesity in offspring, setting the stage for further perpetuation of the intergenerational cycle (Ehrenthal *et al.*, 2013; Galliano & Bellver, 2013). Phelan's study revealed two things about the educational gap between provider and patient: the absence of counseling or possible unwillingness to talk about the topic of weight gain, and a potentially existing knowledge gap within the medically educated population regarding obesity and its reproductive effects (Phelan, 2010; Phelan *et al.*, 2011).

Options to bridge this disparity could involve targeted interventions that increase exposure and public awareness of the subject, leading to increased patient motivation and comfort in discussing the topic of weight management and obesity with their healthcare providers. Our survey participants already demonstrated high knowledge regarding the general health effects of obesity, and a more focused approach on the reproductive effects of obesity might increase this knowledge to a comparable level. In our survey, 58.8% and 60% of educated and medically educated participants, respectively, believed that obesity and reproductive issues information should come from public media. Furthermore, 56.9% and 50.2% of educated and medically educated participants, respectively, believed that schools should also play a role in this topic.

An example of public health campaigns that used similar delivery mechanisms is the tobacco cessation programs. Programs like the truth^®^ campaign have used television commercials to inform young adults of the negative consequences of smoking. These commercials, while at times provocative and graphic, were well received by their target audience, yielding an identifiable decreased risk in smoking initiation (Farrelly *et al.*, 2009). From the perspective of reproductive health, however, it may be more beneficial to modify our approach by emphasizing positive outcomes and the reproductive, maternal, and fetal benefits of losing weight. It may take some time to see measurable effects from a public health campaign, but even the simple act of promoting initial awareness can serve as a promising first step. Therefore, obesity prevention should be one of the most important strategies for any patient who may become pregnant in the future.

Many of our participants believed that women would modify their behavior if they knew about the reproductive risks associated with obesity. This high level of receptiveness, combined with the identified need for increased education in our healthcare-associated survey population, supports the conclusion that the availability of better public health resources and the increased presence of obesity education and nutrition as part of the healthcare curriculum might be suitable next steps towards closing the knowledge gaps in both patients and providers.

Limitations of this study include: small survey group, relatively low response rate, and implementation at only one institution. Future surveys should also gather information on year of medical or residency training, as well as specialty. It is likely that residents in obstetrics, especially in more advanced levels of training, would have more knowledge on this type of survey. Because of the small group and single setting, these results may not be applicable to the general educated population. Larger survey pools and more widespread survey locations would be needed to increase the strength of our findings. A low survey response rate might indicate low general interest in the topic at hand, despite high motivation from those who did complete the survey. Also, the Cardozo study found a statistically significant relationship between education and obesity, where individuals with at least a 4-year college degree were less likely to be obese (Cardozo *et al.*, 2012). Since our population also fits into this educational category, it may be more difficult to draw conclusions about motivation to undergo weight loss strategies and lifestyle changes in a population that is already more likely to have normal weight.

Our study revealed knowledge gaps regarding the reproductive and pregnancy-related health risks of obesity even in an educated lay and medical population. Furthermore, the healthcare-associated component of the population was less knowledgeable of these risks compared to their knowledge of the general health risks. These findings indicated that better public educational resources and increased presence of obesity prevention in the healthcare curriculum might bridge this knowledge gap and lead to positive outcomes in the reproductive and pregnancy-related health of women and infants in the future.

## APPENDIX 1 Reproductive Health Survey

Please respond to the following questions to the best of your knowledge using only information you possessed prior to reading this survey. We are interested in getting the perspectives of both men and women in this survey. Therefore, when answering questions about conception, answer from your own perspective (i.e. if you are male, answer from the viewpoint of a male).

Are you aware that there are health risks associated with obesity?

○ Yes
○ No

How aware of obesity related health risks are you?

○ Unaware
○ Somewhat unaware
○ Somewhat aware
○ Aware

Which of these do you consider risks associated with obesity? (check all that apply)

□ Type II diabetes
□ Fatty liver
□ Increased chance of heart disease
□ Hypertension
□ Sleep difficulties
□ Arthritis
□ Early death
□ Others not mentioned
□ None

How do you personally determine whether or not you consider someone obese?

Do you think that a female who is 5' 9" and weighs 209 pounds is overweight or obese?

○ Yes
○ No

Do you think that a female who is 5' 5" and weighs 180 pounds is overweight or obese?

○ Yes
○ No

Do you believe that weight loss in overweight individuals may help improve the safety of pregnancy and improve fertility?

○ Yes
○ No

Please indicate to what extent you agree or disagree with the following statements.

**Table:**

	Strongly agree	Agree Neither	Neither agree nor disagree	Disagree	Strongly disagree
If people were aware that serious reproductive risks are associated with obesity, they should be motivated to be normal weight or to lose weight before trying to get pregnant.	○	○	○	○	○
If people were aware that serious reproductive risks are associated with obesity, they would actively attempt to lose weight or reach/maintain a normal weight before trying to get pregnant.	○	○	○	○	○

Before taking this survey, were you aware that obesity increases the risk of the following cancers:

**Table:**

	Yes	No
Uterine cancer	□	□
Ovarian cancer	□	□
Postmenopausal breast cancer	□	□

How aware of reproductive problems due to obesity are you?

○ Unaware
○ Somewhat unaware
○ Somewhat aware
○ Aware

Before taking this survey, were you aware that female obesity (as defined by a Body Mass Index (BMI) over 30) increases the risk for:

**Table:**

	Yes	No
Menstrual period irregularity	□	□
Heavy menstrual bleeding	□	□
Lower chances of getting pregnant	□	□
Less responsiveness to fertility treatments	□	□
Higher rate of miscarriages	□	□
Entering puberty at a younger age	□	□

How aware are you of risks to the baby in utero due to maternal obesity?

○ Unaware
○ Somewhat unaware
○ Somewhat aware
○ Aware

How aware are you of risks to the baby after birth due to maternal obesity?

○ Unaware
○ Somewhat unaware
○ Somewhat aware
○ Aware

Before taking this survey, were you aware that maternal obesity (as defined by a Body Mass Index (BMI) over 30) increases the risk for:

**Table:**

	Yes	No
Pre-eclampsia	□	□
Pregnancy diabetes	□	□
Cesarean section	□	□
Increased birth defects	□	□
Early neonatal death	□	□
Stillbirth	□	□
Large infants	□	□

How aware of reproductive issues related to obesity in males are you?

○ Unaware
○ Somewhat unaware
○ Somewhat aware
○ Aware

Before taking this survey, were you aware that certain hormonal birth control methods may be less effective in obese women?

○ Yes
○ No

If you were educated about these types of obesity related facts/risks, would it increase your motivation to be normal weight or to lose weight before trying to conceive a child?

○ Yes
○ No

Would you prefer to get more information about obesity and reproductive issues from (check all that apply):

□ Public media
□ Doctors
□ Schools
□ Other ____________________

Do you think it is possible that maternal obesity in pregnancy may predispose children to obesity?

○ Yes
○ No

Do you have any biological children?

○ Yes
○ No

[If respondent answers 'yes' to "Do you have any biological children?"] Are you planning on having more biological children?

○ Yes
○ No
○ Maybe

[If respondent answers 'no' to "Do you have any biological children?"] Do you plan on having your own biological children someday?

○ Yes
○ No
○ Maybe

In the last year, have you sought out information about fertility issues, pregnancy or related information?

○ Yes
○ No

What is your gender?

○ Male
○ Female

What is your current age?

○ Under 21
○ 21-23
○ 24-26
○ 27-29
○ 30-32
○ 33-35
○ Over 35

What is the highest level of education you have completed?

○ Less than High School
○ High School / GED
○ Some College
○ 2-year College Degree
○ 4-year College Degree
○ Some Graduate School
○ Master's Degree
○ Doctoral Degree
○ Professional Degree (JD, MD)
